# Educational telerehabilitation program for women with pelvic floor dysfunctions after gynecological pelvic cancer treatment: protocol study for a randomized and controlled clinical trial

**DOI:** 10.1186/s13063-024-08138-1

**Published:** 2024-05-28

**Authors:** Tatiana de Bem Fretta, Gabriela Dassie Dacanal, Pauliana Carolina de Souza Mendes, Mirella Dias, Cristine Homsi Jorge

**Affiliations:** 1 Faculty of Medicine of Ribeirão Preto, University of São Paulo, Av. Bandeirantes, 3900, Monte Alegre, Ribeirão Preto, São Paulo CEP: 14049-900 Brazil; 2 Oncology Research Center, Rod. Admar Gonzaga, 655—Itacorubi, Florianópolis, Santa Catarina Brazil

**Keywords:** Brachytherapy, Dyspareunia, Neoplasm, Pelvic floor, Telerehabilitation, Urinary incontinence

## Abstract

**Background:**

Educational and self-care measures are important for women after gynecological pelvic cancer treatment. Pelvic floor muscle training exercises (PFMT) are a conservative treatment for pelvic floor (PF) dysfunction. The purpose is to evaluate the impact of a telerehabilitation and self-care program on PF dysfunctions, reports of urinary incontinence (UI), and physical–emotional factors of participants post-treatment for gynecological pelvic cancer.

**Methods:**

Two-arm randomized clinical trial: an intervention group (IG) will evaluate the effect of a telerehabilitation program on women undergoing clinical practice of radiotherapy for the treatment of gynecological pelvic cancer and a control group (CG) will maintain the routine. Primary outcome is the prevalence of reports of UI, which will be assessed using the International Consultation on Incontinence Questionnaire—Short Form (ICIQ-SF). The secondary outcomes will be the severity and impact of UI on quality of life, location and perception of pain intensity, presence and intensity of dyspareunia, vaginal stenosis, fecal incontinence (FI), and levels of physical activity. Statistical analysis will be performed by intention-to-treat, and multivariate mixed effects analysis will be used to compare results.

**Discussion:**

Activities in the context of telerehabilitation using PFMT and self-care can represent a viable and effective solution to minimize the side effects of gynecological cancer treatment and improve women’s quality of life.

**Supplementary Information:**

The online version contains supplementary material available at 10.1186/s13063-024-08138-1.

## Background

Advances in treatment for gynecological pelvic cancer have improved women’s survival, but as a side effect, the risk of pelvic floor (PF) dysfunction has increased [1, 2]. A systematic review showed that the main PF dysfunctions after treatment of gynecological neoplasms are urinary incontinence (UI), fecal incontinence (FI), and sexual dysfunctions [3]. Other PF dysfunctions include vaginal stenosis [4], pelvic pain [5], and decreased muscle function [6]. This type of cancer sensitizes a woman’s feminine identity, as it causes sexual dysfunctions [5, 7–9], dyspareunia [6–8], low self-esteem [8, 10], decreased quality of life [7, 8], and decreased physical activity [11, 12].

It is found in the literature through a systematic review that pelvic floor muscle training exercises (PFMT) is the first line of treatment for UI [13], in improving sexual function [14, 15], and in vaginal stenosis [16]. PFMT is defined as an exercise to improve strength, endurance, power, and relaxation or a combination of these factors [17]. It is certified in the literature on telerehabilitation using PFMT in the treatment of PF dysfunctions and promoted a significant improvement in urinary symptoms, PFM function, and quality of life [18].

Telerehabilitation has become increasingly common, the availability and accessibility of care for women who live far from rehabilitation centers becoming increasingly viable [19]. It is important to highlight that telerehabilitation has educational and self-application objectives that require the development of educational support material that is suitable for the virtual environment. Educational videos and applications have great potential to contribute to virtual care, but there are few materials of this type specifically designed for this purpose that have been tested for their usefulness prioritizing a physical activity plan and self-care measures to contribute to a better quality of life for women [20].

Considering that cancer pelvic is currently among the health problems in the world, and its treatment contributes to PFM dysfunctions such as UI, it is urgent to investigate in high-quality RCTs new low-risk interventions capable to optimize the results obtained through telerehabilitation. Thus, this protocol study aims to evaluate the impact of a telerehabilitation and self-care program on PF dysfunctions, reports of UI, and physical–emotional factors of participants post-treatment for gynecological pelvic cancer.

## Methods

### Study design

Randomized and controlled, two-arm blinded evaluator clinical trial was designed to evaluate the effect of a telerehabilitation program in women undergoing clinical practice of radiotherapy and brachytherapy of gynecological pelvic cancer on the primary outcome of the prevalence of reports of UI, which will be evaluated using the following question from the International Consultation on Incontinence Questionnaire—Short Form (ICIQ-SF) “how often do you lose urine?”.

Secondary outcomes will be the severity and impact of UI on quality of life, sexual function, FI, self-esteem, quality of life, location and perception of pain intensity, presence and intensity of dyspareunia, vaginal stenosis, and physical activity levels. Finally, the intervention group will respond about treatment satisfaction, adherence to supervised sessions, home guidance through home diary recording, recording of adverse effects, and System Usability Scale.

Participants will be randomized to an intervention group that will participate in a telerehabilitation program or control group. This protocol will be reported according to the Protocol Items Standard: Recommendations for Intervention Trials checklist (SPIRIT) 2013: items recommended for a clinical trial protocol and related documents. It is a superiority trial.

### Ethic

This study will be carried out in accordance with the Declaration of Helsinki (1975) and was approved by the Ethics Committee for Research with Human Beings (CEPSH) of the Hospital das Clínicas of the Faculty of Medicine of Ribeirão Preto under protocol no. 4,718,873 and Committee Ethics Committee of the Oncology Research Center (CEPON) under protocol no. 5,013,364 and registered in the Brazilian Clinical Trials Registry (ReBEC) no. RBR-8ht5nqq.

For ethical reasons, we did not plan to prohibit any concomitant treatment. We included in the “Methods” section our plan to monitor any concomitant treatment for PF dysfunctions including UI, but we expect to have homogeneous groups in relation to these variables provided by an adequate randomization and sample size.

**Fig. 1:**
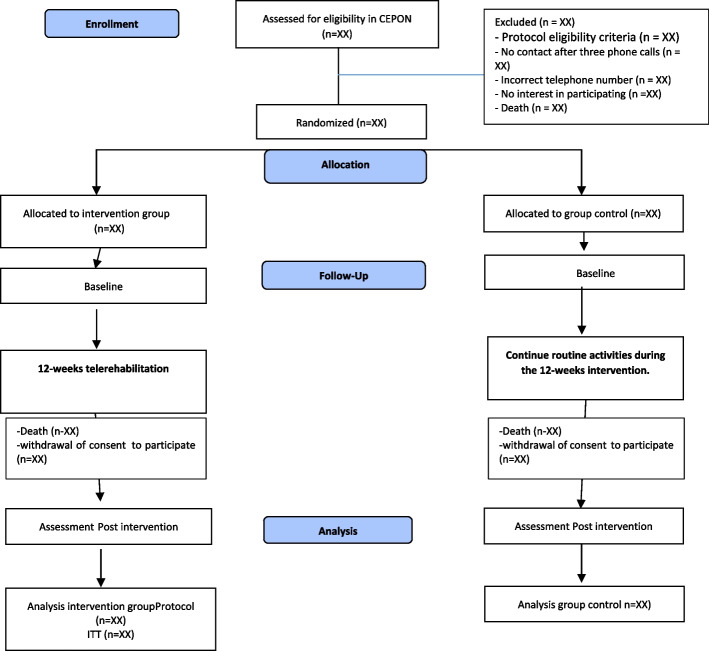
The flowchart for selecting women for the randomized clinical trial, following the CONSORT

**Fig. 2:**
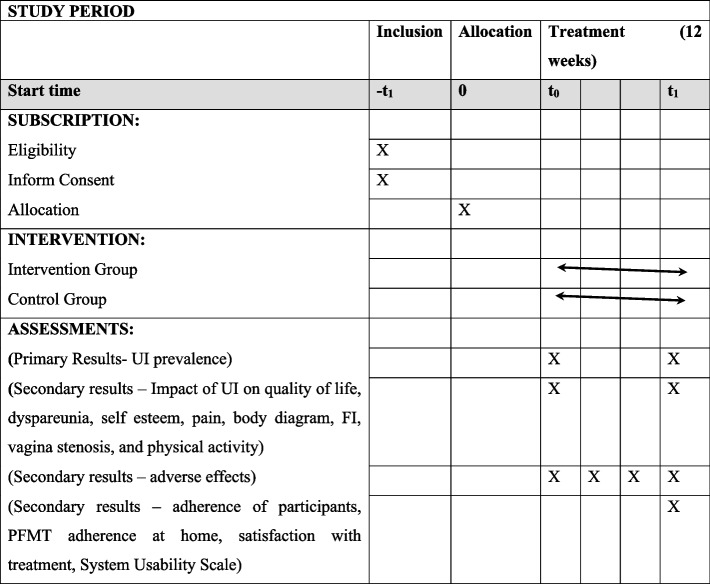
Study evaluation schedule (SPIRIT). Source: According to the SPIRIT 2013 statement: Standard Protocol Item Definition for Clinical Trials

Figure 1 shows the CONSORT (Consolidated Standards of Reporting Trials) flowchart, enrollment schedule, interventions, and study evaluations. Figure 2 shows the checklist using the SPIRIT (Standard Protocol Items: Recommendations for Interventional Trials) used in the study (Additional file 1).

### Participants

The study will consist of participants diagnosed with gynecological pelvic cancer living in the State of Santa Catarina who underwent treatment at the Oncological Research Center—CEPON in Florianópolis, Santa Catarina, Brazil.

The principal investigator (TBF) will have access to a telephone list of women who have completed radiotherapy and/or brachytherapy treatment for gynecological pelvic cancer in the years 2020 to 2022. This researcher will contact these participants by telephone and ask “how often do you lose urine?” Participants with UI will receive an explanation of the stages of the study, and after consenting to participate, they will sign a consent form and then be taken for an individual interview with a scheduled day and time for data collection via google meeting; these interviews will be carried out with the researchers (GDD or PCSM). In addition to the institution’s routine procedure, a physiotherapy session every 3 months for 5 years is included. During these sessions, the physiotherapist provides guidance on PFMT and the use of vaginal dilators.

### Eligibility criteria

The study will consist of participants with UI after radiotherapy and/or brachytherapy for gynecological pelvic cancer who underwent treatment at the Oncological Research Center—CEPON in Florianópolis, Santa Catarina, Brazil.

Women are defined as inclusion criteria: age group over 18 years old; clinical stages I to III of gynecological pelvic cancer; having undergone surgery, radiotherapy, and/or brachytherapy; reporting stress UI, urgent UI, or mixed UI (self-reporting by the question Short Form (ICIQ-SF) “how often do you lose urine?”); do not have orthopedic or neurological limitations that prevent the practice of the telerehabilitation program; and not undergoing physiotherapeutic treatment.

### Criteria of discontinuing

The criteria of discontinuing the trial for a given participant include withdrawal of consent to participate.

### Sample size

G*Power 3.1.9.2 software was used to calculate the sample size based on the difference between two means using the *T*-test, after a 12-week pilot study with 20 participants, 10 in the control group (CG) (2.8 ± 1.9) and 10 in the intervention group (IG) (1.41 ± 1.50). The variable considered for the calculation was the prevalence of UI through the ICQ (short-form) question “how often do you lose urine?” The answer is presented in the form of a Likert scale.

Taking these observations into account, a total number of 58 participants (29 IG and 29 CG) will be needed to detect a difference between the groups in the ICQ-SF question “how often do you lose urine?” after 12 weeks of intervention, assuming an alpha level of 0.05 and 80% power of the test.

### Statistical analysis

Data will be de-identified, encoded, and stored in the database using the Excel Program. The software SPSS 22.0 will be used for the analysis of data. The description of quantitative variables will be expressed in mean, standard deviation, minimum value, median, and maximum value. Qualitative variables will be described in absolute and relative values (%). For all primary and secondary results, a 95% confidence interval and a significance level of 0.05 will be considered in the test. Both per-protocol and intention-to-treat analysis will be performed.

A linear mixed-effect model will be used to analyze the ICIQ-SF score, FSFI score, FIQL score, EORTC score, IPAQ score, ROSEMBERG score, and System Usability Scale. In the reassessment, individuals’ values will be considered random effects, and the groups, the times, and the interaction between them will be regarded as fixed effects. In addition, the McNemar test will verify changes in categories variables before and after the intervention. Both per-protocol analysis [21–23] and an intention-to-treat analysis will be also conducted. The Cohen effect size (*d*) will be calculated using the averages.

### Randomization and blinding process

A researcher specially trained will be responsible only for recruiting the participants to enter the study and will give them all the information about the study, obtain your consent through Google Forms.

An assistant researcher not involved with the recruitment, intervention, and assessment will generate the allocation sequence. The participants will be allocated to the following groups: A—intervention, an educational program of telerehabilitation and PFMT practice and B—control, who will be invited to maintain their routine activities. Randomization will be carried out via website (http://www.randomization.com) using a computational list of random numbers and will be concealed (by means of a sealed brown envelope).

Participants’ data will be kept only with the assistant researcher not involved with other parts of the research process to process to protect confidentiality before, during, after the intervention, and during data analysis. The trial is assessor-blinded in relation to the allocation of the participants.

The senior researcher who is not involved with the recruitment, intervention delivery, and assessment will have access to the trial data. We have planned to submit the manuscript to an open access journal that make all data available to readers as an appendix or under request.

### Intervention

Participants allocated to the intervention group will participate in an educational program, consisting of 12 consecutive 90-min meetings offered once a week for 12 weeks, which will be held virtually via Google Meet. The intervention will be conducted by a physiotherapist with 23 years of training and who is the main researcher.

The telerehabilitation program will include lectures on the anatomy, functions, and dysfunctions of the PF muscle, risk factors for the development of disorders, therapeutic options, and preventive measures, with a strong focus on the PF muscle through exercises and awareness of the function of the PF muscle during different tasks of daily life that increases intra-abdominal pressure, such as coughing, sneezing, and other activities and urinary urgency.

To carry out the didactic activities, illustrative figures from the PFMT will be used. The lectures will include topics such as gynecological pelvic cancer, physical activity and cancer, UI, continence mechanism, vaginal stenosis, sexual dysfunction, dyspareunia, FI, and sexual dysfunctions after treatment for gynecological pelvic cancer, and all meetings will have a practical part with supervised PFMT. After the lectures are finished, the material used in the lectures will be sent by e-mail.

The intervention protocol is described in detail in Table 1, and Table 1 contains the PFMT progressions. A record of adherence to supervised sessions will be made, as well as a record of adverse effects during the 12 weeks of intervention.

**Table 1:** PFMT progression parameters

Sustained contractions															
**Week**	**No. of series**		**Repetitions**			**Contraction holding time**						**Relaxation time**			
1	2		6			4 s						4 s			
2	2		8			4 s						6 s			
3	2		10			5 s						8 s			
4	2		10			5 s						10 s			
5	2		10			6 s						10 s			
6	1		10			6 s						10 s			
7	1		10			7 s						10 s			
8	1		10			8 s						10 s			
9	1		10			9 s						10 s			
10	1		10			10 s						10 s			
11	1		10			10 s						10 s			
12	1		10			10 s						10 s			
**Fast contractions**															
**Week**		1		2	3	4	5	6	7	8	9		10	11	12
**No. of repetitions**		6 ×		6 ×	6 ×	10 ×	10 ×	10 ×	15 ×	15 ×	15 ×		20 ×	20 ×	20 ×

Progression of the 12 weeks of PFMT

**Frame 1** Roadmap for carrying out educational activities

**Table:** 

1st meeting	Presentation of the team, justification for the group’s existence, and proposals regarding the health of women with gynecological pelvic cancer; What will be covered in the group; Explain the anatomy and physiology of the PF and genitourinary system: use of figures and computer-animated models; explanation about the stop test that will be carried out at home Stop test; Dynamics for body self-knowledge: activity on how to contract the PF, abdominal breathing technique for relaxation; Practice PFMT in the four positions sitting: lying down, four supports, sitting, and standing
2nd meeting	Physical activity and cancer; guidelines; aerobic and resistive exercises; authorization from the doctor to practice physical activity; Physical activity after gynecological pelvic cancer treatment; Benefits of physical activity in PF; Care; Stretching exercises Practice PFMT in the four positions sitting: lying down, four supports, sitting, and standing
3rd meeting	Gynecological pelvic cancer (types and etiology) Risk factors; Treatment; PF dysfunctions after cancer treatment; Physiotherapy; PF awareness, proprioception, and contraction exercise; Practice PFMT in the four positions sitting: lying down, four supports, sitting, and standing Practice breathing;
4th meeting	Impact of UI on quality of life; UI types; Explanation about PFMT and “the Knack” Practice PFMT in the four positions sitting: lying down, four supports, sitting, and standing
5th meeting	Continence mechanisms; Risk factors for UI in women and after treatment for gynecological pelvic cancer; Problem prevention: importance of prevention due to high prevalence; PF exercises, proprioception and contraction exercises; Practice PFMT in the four positions sitting: lying down, four supports, sitting, and standing; Practice breathing
6th meeting	Exams and possible treatments for UI; Role of physiotherapy in the process of comprehensive health: techniques and devices; Stretching exercises Practice PFMT in the four positions sitting: lying down, four supports, sitting, and standing
7th meeting	Vaginal stenosis after gynecological pelvic cancer treatment; Impact of vaginal stenosis on quality of life; Impact of sexual dysfunction vaginal stenosis; Treatment and encouragement of the use of vaginal dilators; Perineal massage; Practice PFMT in the four positions sitting: lying down, four supports, sitting, and standing; Practice breathing
8th meeting	Sexual dysfunction after gynecological pelvic cancer treatment; Dyspareunia; Impact of sexual dysfunction on quality of life; Practice PFMT in the four positions sitting: lying down, four supports, sitting, and standing
9th meeting	Fecal incontinence after treatment for gynecological pelvic cancer; Impact of anal incontinence on quality of life; Abdominal massage; Practice PFMT in the four positions sitting: lying down, four supports, sitting, and standing; Practice pelvic mobility exercises
10th meeting	Self-care in women’s health; Practice PFMT in the four positions sitting: lying down, four supports, sitting, and standing; Practice breathing
11th meeting	Resumption of main concepts; Practice PFMT in the four positions sitting: lying down, four supports, sitting, and standing;
12th meeting	Free; Practice PFMT sitting, standing, performing squats, bridges, and walking. Dance for pelvic mobility

Description of the stages of the 12 meetings

A Whatsapp group will be created with the participants and the main researcher as a way of welcoming the project, monitoring, and encouraging these participants to carry out PFMT at home, use vaginal dilators, and practice physical activity. In infographic 1, examples of the folder will be sent to the participants one times a week.

**Infográfico 1:** Folders will be sent to participants throughout the 12 weeks.
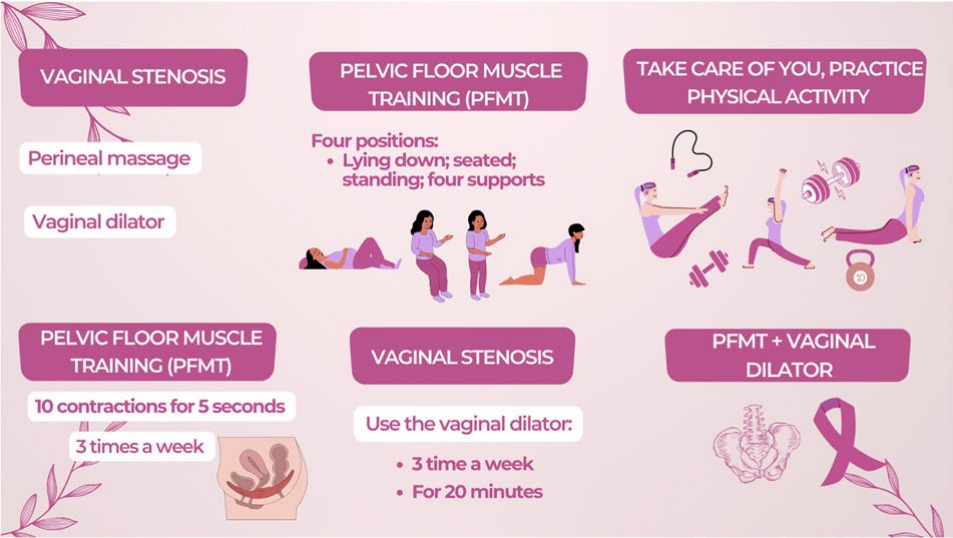


During the 12 weeks of the study, participants will be monitored on their adherence to perform the PFMT at home. They will complete a daily record booklet on how many contractions they performed, ranging from less than 10 contractions per day, from 10 to 20 contractions per day, from 20 to 30 contractions per day, from 30 to 40 contractions per day, and more than 40 contractions per day. Consequently, how many times a day, the contractions will take place, one time, two times, three times, or more than three times a day, as well as the positions in which the contractions will take place lying down, sitting, on all fours, and standing. In infographic 2, it shows how it will constitute the primer.

**Infographic 2:** Booklet for daily recording of the PFMT.
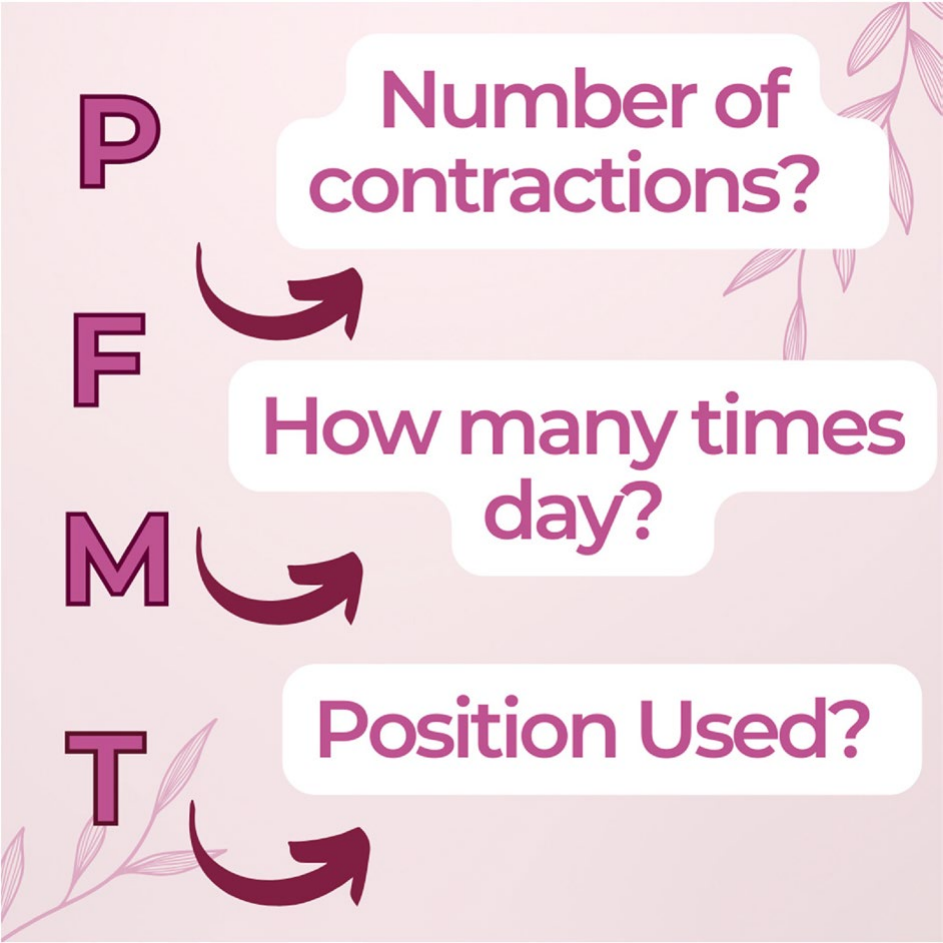


Participants will fill out a diary about their adherence to PFMT, and there will be a place where they will be asked about any side effects or discomfort related to the intervention. At the last assessment, they are asked again about any unintended effects related to the experimental intervention. The type and number of undesirable effects will be reported in full as an important outcome of the trial. Although adverse effects from these types of interventions are not very common or serious, all participants are assured that they can discontinue their participation at any time for any reason, including any adverse effects.

### Group control

Participants in the CG will be invited to maintain their routine activities and will have the opportunity to participate in the telerehabilitation program after the 12 weeks that served as control, in a second moment that will not be for research purposes, since the results could be influenced by previous assessments and reassessments.

### Assessment

#### Primary outcome measures

The primary objective of this study will be to evaluate the impact of a PF telerehabilitation program on the prevalence of reports of UI in participants post-radiation therapy treatment for gynecological pelvic cancer. UI is the primary outcome at all times of the study, that is, baseline and after 12 weeks of intervention. Therefore, could the intervention of an educational telerehabilitation program promote improvements in reports of UI in these participants?

The prevalence of UI will be assessed using the following question from the International Consultation on Incontinence Questionnaire—Short Form (ICIQ-SF). “How often do you lose urine” being 0—never, 1—once a week or less, 2—two or three times a week, 3—once a day, 4—several times a day, and 5—how long all.

#### Other outcome measures

##### The severity and impact of UI on quality of life

It will be assessed by the ICIQ-SF questionnaire; this questionnaire was translated into Brazilian Portuguese and culturally validated by Tamanini et al. [24] and consists of six questions with the aim of evaluating UI frequency, its perceived cause, severity, and its impact on quality of life. These will be measured using the total ICIQ-SF score (sum of questions one, two and three); the scores for this instrument range from 0 to 21 points, where higher scores indicate greater severity.

##### Sexual function

Sexual function will be assessed using the Female Sexual Function Index (FSFI) with cross-cultural validation [25], revealing a Cronbach’s *α* of 0.96. This questionnaire consists of 19 questions grouped into six areas: desire, arousal, lubrication, orgasm, satisfaction, and pain. The sexual function score at the end of the analysis can vary from 2 to 36 points, and the higher the score obtained, the better the woman’s sexual function.

Values equal to or below 26.55 in the general score are considered indicative of sexual dysfunction. There is also a cutoff point for each domain as follows: desire ≤ 4.28, arousal ≤ 5.08, lubrication ≤ 5.45, orgasm ≤ 5.05, satisfaction ≤ 5.04, and pain ≤ 5.51 [26].

##### Dyspareunia

The intensity of dyspareunia will be used on a numerical assessment scale from 0 to 10, with 0 representing “no pain” and 10 the “worst pain imaginable” [27].

##### Self-esteem

The Self-Esteem Scale developed by Rosenberg [28] will be used. This scale was validated for the cancer population [29] and in Brazil [30]. It also received a validation review [31] with Cronbach’s *α* of 0.90. It is a unidimensional measure composed of ten statements related to a set of feelings of self-esteem and self-acceptance that determine global self-esteem. The total scale score varies from 10 to 40 points, being used for categorization: (1) satisfactory or high self-esteem, those with a score greater than 31 points; (2) average self-esteem, total score between 21 and 30 points; and (3) unsatisfactory or low self-esteem, with a score below 20 points. It is understood, therefore, that the higher the value achieved by the woman on the scale, the better her self-esteem.

##### Pain

A numerical scale from 0 to 10 will be used to assess pain, with 0 representing “no pain” and 10 the “worst pain imaginable” [27]. Participants will receive a body diagram to identify the topography of the pain.

##### Fecal incontinence

A validated and culturally adapted self-report questionnaire will be used [32], the Fecal Incontinence Quality of Life (FIQL) questionnaire, which is specific to evaluating the impact of FI on quality of life, the results for intra- and inter-examiner reproducibility showed agreement significant in all domains of the questionnaire, Kappa coefficient lifestyle 0.93, behavior 0.93, depression 0.95, and embarrassment 0.92. It contains 29 questions, which allow calculating impact scores on quality of life on four scales: lifestyle, behavior, depression, and embarrassment. The lower the score on each scale, whose score can vary from 1 to 5, the lower the functional status of quality of life.

##### Bristol stool consistency scale

To assess the consistency of feces, adapted for Brazil [33], this Bristol scale has been recognized by scientific literature as a valuable tool in the assessment of intestinal diseases. The general Kappa index was 0.826; the scale demonstrates high reliability in all groups studied.

##### Vaginal stenosis

It will be by self-report using the common terminology criteria for adverse events v.4.0 9 (CTCAE) [34] stenosis classification. On this scale, stenosis is classified into three grades: grade 1 is characterized as asymptomatic shortening or narrowing, grade 2 is shortening or narrowing that does not interfere with physical examination, and grade 3 is shortening or narrowing that interferes with the use of tampons, relationship sexual examination, and physical examination [31].

##### Quality of life

European Organization for Research and Treatment of Cancer Quality of Life Questionnaire C30 (EORTC QLQ-C30). This instrument was created by the European Organization for Research and Treatment of Cancer (EORTC) in 1986, and this is the third version [35]. Validated for the cancer population by Michels et al., [36] Cronbach’s *α* values were 0.72 for global health, 0.86 for the functional scale, and 0.81 for the symptomatic scale.

The instrument consists of 30 questions, being multidimensional and self-administered. Its objective is to evaluate the quality of life in cancer patients in the last 4 weeks. It features five functional scales (physical, functional, emotional, social, and cognitive), a global health status scale, three symptom scales (fatigue, pain, and nausea/vomiting), and six additional symptom items (dyspnea, appetite, constipation, diarrhea, and financial difficulties). The answers are presented in the form of a Likert scale following the classification 1 = not at all, 2 = a little, 3 = a lot, and 4 = a lot. The only exception applies to the global health scale. This consists of two questions that ask the patient to evaluate their health and quality of life in the last week according to scores from 1 to 7.

##### Level of physical activity

The level of physical activity will be investigated using the International Physical Activity Questionnaire (IPAQ—short version) [37]. Brazilian validation and reproducibility were carried out by Matsudo [38] and had a significant Spearman correlation and high reproducibility (rho = 0.69–0.71; *P* < 0.01) and validity of 0.75. It consists of six items that seek to verify the number of times that the subject practiced at least ten continuous minutes of walking, moderate and vigorous physical activity, in the last week, in different involvements, namely work, domestic, leisure, recreational, and sports. After completing the questionnaire, participants can be classified into the categories of sedentary, insufficiently active, and very active. The IPAQ also addresses individuals’ sitting time during the week and on weekends. There are two specific questions that ask: (1) How much time in total do you spend sitting on a weekday? and (2) How much time in total do you spend sitting on a weekend day? Data is presented in minutes per week.

##### Treatment satisfaction

Using a numerical scale, it will be composed of a unidimensional measure to assess treatment satisfaction. Composed of a line from 0 to 10 with anchors at both ends, at one end of the line is marked 10 “completely satisfied” and at the other 0 “dissatisfied”.

##### Home guidelines

A home diary will be delivered to record the PFMT; in this record, the participant will record the number of contractions performed, how many times a week the PFMT was performed, and the position used (infographic 2).

##### Adherence to supervised sessions and recording of adverse effects

A record will be made by the main researcher during the intervention regarding adherence to supervised sessions, as well as adverse effects during the intervention through a diary record.

##### System Usability Scale (SUS)

Post-intervention, participants will investigate the usability of the system used; the System Usability Scale (SUS) instrument contains ten questions that are grouped on a Likert scale, with values from 1 to 5, classified respectively as: “strongly disagree,” “disagree,” “neither agree nor disagree,” “agree,” and “strongly agree”. Only the last question, included by the researcher, was a non-mandatory essay. The estimated total time to answer the questionnaire was 5 to 10 min. After completing the SUS, the total score was calculated, which generated a unique number. To calculate the score, the score of each contributing item is first added together on a scale of 1 to 5. For items 1, 3, 5, 7, and 9, the individual score is the grade received minus 1. For items 2, 4, 6, 8, and 10, the contribution is 5 minus the grade received. The sum of all scores is multiplied by 2.5 and the total SUS value is obtained. After scoring and calculating the score, it is possible to classify the evaluated system: 20.5 (worst imaginable); 21 to 38.5 (poor); 39 to 52.5 (median); 53 to 73.5 (good); 74 to 85.5 (excellent); and 86 to 100 (best imaginable) [39, 40].

##### Descriptive and control variables

The descriptive and control variables were divided into clinical variables (cancer stage, treatment characteristics, previous clinical treatment chemotherapy, teletherapy and brachytherapy, characteristics of the surgical intervention, date of surgery, gynecological–urological history, other diseases, smoker, and use of alcohol), sociodemographic variables (age, education, marital status, economic level, and occupation), and anthropometric measurements (height and body mass). Descriptive and control variables will be acquired by self-report. The study variables referring to the intervention protocol are presented in Table 2.

**Table 2:** Outcome measures and time points the study assessment

Results	Instruments	Baseline	Post-intervention
Urinary incontinence and quality of life	International Consultation on Incontinence Questionnaire—Short Form (ICIQ-SF)	√	√
Function sexual	Female Sexual Function Index (FSFI)	√	√
Self-esteem	Self-esteem scale Rosenberg	√	√
Pain	Numerical pain scale	√	√
Body diagram	Topography of pain	√	√
Dyspareunia	Numerical pain scale	√	√
Fecal incontinence	Fecal Incontinence Quality of Life (FIQL)	√	√
Stool consistency	Bristol stool consistency scale	√	√
Quality of life	European Organization for Research and Treatment of Cancer Quality of Life Questionnaire C30 (EORTC QLQ-C30)	√	√
Vagina stenosis	Common terminology criteria for adverse events v.4.0 9 (CTCAE)	√	√
Physical activity level	International Physical activity questionnaire (IPAQ—short version)	√	√
Treatment satisfaction	Numerical pain scale		√
System Usability Scale	(SUS)		√

### Data collection

Data collection will be previously scheduled with participants online via Google Meet. The questionnaires will be applied in an interview format and will last 60 min; a researcher will receive training beforehand for data collection and will not participate in the intervention. The questionnaire covers general and clinical information, UI, sexual function, self-esteem, pain, dyspareunia, FI, and quality of life. All data collection will take place in two moments, firstly, in the period before the start of the intervention, called baseline (T0), and at the moment after the first stage of the intervention, that is, after the initial 12 weeks (T1) (Fig. 1).

Data collection from the control group will be carried out using the same questionnaires applied to the intervention group. Collection will be scheduled with the participants and will take place at the same interval as the intervention group.

## Discussion

When planning this protocol, we considered what has already been proven in the literature to optimize the effectiveness of PFMT in PF dysfunctions caused by gynecological pelvic cancer treatment. It is essential to offer a high-quality intervention based on the physiology and morphology of the PFM. It was designed following quality criteria to increase internal and external validity.

Activities in the context of telerehabilitation using PFMT and self-care can represent a viable and effective solution to minimize some side effects of gynecological cancer treatment and improve women’s quality of life. Although some studies have already evaluated aspects of PFMT for UI [41] and vaginal stenosis [16] after gynecological pelvic cancer, there is a great need for the development and implementation of treatment models. Telerehabilitation can be offered by the public system, in addition to being low cost for rehabilitation centers. All techniques used in this protocol represent a minimum risk of adverse effects, facilitating the acceptance of educational measures with PFMT for this population.

Educational programs provide information, health education, and self-care and well-being practices, with a focus on health care. According to the Health Belief model [42], health education aims to inform, modify behaviors, identify gaps in knowledge, offer information, motivation, or concern about health, understand the sequelae of the disease, and overcome perceived barriers.

An educational program of four group meetings offered guidance on the functions, dysfunctions of the PF muscles, and therapeutic options with an emphasis on conservative treatment through PFMT. In this randomized and controlled clinical trial, women were also instructed to perform “the Knack” maneuver while carrying out activities of daily living. A significant improvement in women’s knowledge about the location, functions, and dysfunctions of the PF was observed [43]; however, there was no significant improvement in UI. The study demonstrated the usefulness of this format of educational activity to inform the female population in general, indicating, however, the need for specific PFMT programs when the objective is to rehabilitate the PF muscles and treat UI.

Cochrane’s systematic review [19] reinforced that telephone interventions have the potential to provide supportive information and promote changes in behavior, adherence to treatments, and self-care. These interventions have benefits as they reduce the severity of symptoms, thus improving patients’ quality of life. The use of telerehabilitation in women with UI using PFMT as a treatment has already been discussed in the literature. Telerehabilitation was as effective as in-person care; being a new treatment, promising, and effective with the advancement of technology, it is a tool in communication between physiotherapists and patients [18].

Another important aspect is PFMT in sexual dysfunctions; vulvovaginal toxicity after radiotherapy treatment causes in PF: vaginal dryness, vaginal stenosis, and dyspareunia [6–8]. PFMT aims to improve the functionality of the PF by improving the contractile response and the ability to perform maximum force; thus, PFMT is a treatment that can be effective in improving sexual function and reducing dyspareunia [6–8].

The constant evolution of cancer treatments has provided an increase in the survival time of these women, which has led to a new focus of research, in an attempt to reduce the side effects of treatments and help these women return to their routine activities. In this context, physical activity reduces the effects caused by treatment, reduces mortality, and cancer recurrence, becoming a possible and effective alternative for complementary treatment for cancer patients [44]. The benefits of physical activity with cancer patients, during and after treatment, in improving cardiorespiratory fitness, fatigue, physical function, quality of life, increased muscle strength, and muscle mass stand out in the literature through a systematic review and meta-analysis [12].

Digital practice is not a modality used in all countries and depends on local regulations. In Brazil, the regulation for telerehabilitation was designed by the Federal Council of Physiotherapy and Occupational Therapy (COFFITO) through Resolution No. 516 of March 20, 2020. In 2017, the World Confederation for Physical Therapy (WCPT) began a collaboration to provide guidelines and regulations related to Digital Physiotherapy and, according to the WCPT Digital Physiotherapy, was defined as a term used to describe services, support, and health information provided remotely through digital devices and communications.

To date, no clinical trial protocols on education and self-care for women after treatment for gynecological pelvic cancer have been found in the literature. This clinical trial is innovative because it investigates the impact of a telerehabilitation model on the side effects caused by gynecological pelvic cancer treatment. It will also investigate the barriers and facilitators to using a telerehabilitation model.

The present protocol can corroborate the literature on the investigation of the effects of a telerehabilitation program of self-care measures associated with PFMT on PF dysfunctions in women after treatment for gynecological pelvic cancer. Upon completion of this research, results will be presented in journals through peer-reviewed publications and scientific events, including the International Congress.

### Supplementary Information

Additional file 1: SPIRIT checklist

## Data Availability

Data are available from authors upon request.
